# Influence of Chronotype and Theobromine on the 24-h Variation in Peak Expiratory Flow Rate in Healthy Adults

**DOI:** 10.5334/jcr.242

**Published:** 2025-04-03

**Authors:** Anika Köhlmoos, Manuela Dittmar

**Affiliations:** 1 Christian-Albrechts-University, Zoological Institute, Human Biology, Germany

**Keywords:** peak expiratory flow, circadian rhythm, early chronotypes, late chronotypes, theobromine

## Abstract

The influence of morningness-eveningness preference and dietary components on the circadian variation in peak expiratory flow (PEF), an established criterion for pulmonary function, is not fully understood. This study aimed to investigate (a) how the chronotype influences the 24-h variation in PEF and (b) whether the bronchodilator theobromine affects this variation differently in different chronotypes. Ninety-seven healthy non-smoking females (54 early chronotypes, ET; 43 late chronotypes, LT; 18–35 years) recorded their PEF over 24 hours at 4-h intervals (08:00/12:00/16:00/20:00/24:00/04:00/08:00 h). In a subgroup (26 ET, 18 LT), the effect of 48 mg theobromine (40 g dark chocolate) on PEF was compared on three consecutive 24-h days with no administration, morning administration and evening administration of theobromine. Repeated measures ANOVA tested for 24-h variation in PEF. Both chronotypes displayed significant 24-h variation in PEF (*P* < 0.0001) explaining 36% (ET) and 31% (LT) of variance of PEF. The time of maximum PEF was three hours earlier in ET than in LT (*P* = 0.003) and correlated negatively with chronotype scores (*P* = 0.001) and positively with bedtimes (awakening time: *P* < 0.001; sleep-onset time: *P* = 0.012). The chronotypes showed no differences in 24-h mean and amplitude % mean for PEF. Administration of theobromine strengthened the morning increase (*P* = 0.004) and weakened the evening decrease (*P* = 0.063) of PEF in LT, but had no effect in ET. The differences found between chronotypes in timing of maximum PEF and responsiveness to the bronchodilator theobromine might have clinical relevance. Therapy for respiratory diseases should consider the chronotype of patients for drug timing and dosage.

## Introduction

Human pulmonary function can be characterized by various parameters. One parameter is the peak expiratory flow rate (PEF), which is a measure of ventilatory capacity of the lung. It reflects the large airway flow through the lung and describes the maximum volume of air that can be achieved per minute during a forced expiration. The PEF depends on age, sex, body height and smoking status [1,2,3,4] and is lower in patients with chronic inflammatory disorders of airways [5,6].

Like many other processes in the human body, the PEF follows a circadian rhythm that exhibits the minimum value during sleeping hours and a maximum value during waking hours. Laboratory studies in normal and asthmatic individuals demonstrated that the circadian minimum for PEF occurred around 04:00 h, followed by a gradual rise during daytime before reaching a maximum PEF around 16:00 h in the afternoon and thereafter gradually decreasing in the evening and at night before reaching the next minimum [7,8]. Most ambulatory diurnal studies in normal and asthmatic individuals, which did not perform PEF measurements during sleeping hours, found a similar pattern, characterized by a minimum PEF around 05:00 h and a maximum PEF around 17:00 h [9,10,11,12].

Interestingly, the described 24-h pattern in PEF does not apply to all humans. A diurnal study found that only 55% of study participants displayed a maximum PEF in the afternoon and 69% a minimum PEF in the early morning [11]. In addition, another study that analyzed diurnal variability in PEF for three days found that only 58% of study participants had the described pattern for PEF [9]. A laboratory study using a constant routine protocol noted prominent and significant individual circadian rhythms for PEF, while the group mean failed to reach significant circadian variation [13]. Henderson and Carswell [14] reported a bimodal distribution of acrophases (time of peak of rhythm cycle) for PEF among normal individuals. It can therefore be assumed that the reported interindividual variation in PEF would reflect the presence of different chronotypes within the study groups. Chronotypes are categorized in early, intermediate and late chronotypes who differ in the timing of their endogenous circadian rhythms and therefore exhibit maximum values at different times. Early chronotypes typically have sleep-wake patterns, objective sleepiness and circadian phase markers for temperature and melatonin two to three hours earlier than late chronotypes [15]. Because early and late chronotypes differ in their sleep-wake times we would expect that early chronotypes with earlier bedtimes would also show an earlier maximum PEF compared to late chronotypes. However, this has not yet been investigated.

The mechanisms that cause the 24-h variation in PEF are processes under circadian regulation and sleep-related physiological changes in respiratory function [16,17]. Sleep increases the parasympathetic tone and the parasympathetic innervation in airways that leads to a higher airway resistance [18,19] and thus to a lower nocturnal PEF. The circadian system significantly modulates pulmonary function independent of sleep. This has been demonstrated by a laboratory study that applied a constant routine protocol with continuous wakefulness and a 196-h forced desynchrony procedure [17]. Circadian changes in autonomic tone, noradrenergic and cholinergic nervous system [20] and inflammatory mediators [21] are potential mechanisms that contribute to the circadian variation of airway resistance measured as PEF.

In addition to circadian and sleep-related factors, there are environmental and behavioral factors that influence airway resistance. Exercise, daytime napping and consumption of certain foods and drinks that contain caffeine and theobromine can briefly induce bronchodilation, thereby reducing resistance in the respiratory airways and increasing airflow through the lungs that results in a higher PEF [22,23,24]. Theobromine (3,7-dimethylxanthine) is particularly interesting because it occurs in various foods and drinks. It is the primary alkaloid of cocoa and its highest proportion in foods is found in dark chocolate. Peak concentrations of theobromine absorption from chocolate occurred in blood plasma approximately two hours after administration [25]. The half-life of theobromine in blood plasma is 6.1 hours in normal volunteers [26] and the bioavailability approximates 96%. Simons et al. [23] demonstrated the bronchodilator effect of theobromine in humans. After administration of a 10 mg/kg dose of theobromine, the peak bronchodilation was reached two hours later and the bronchodilation lasted for six hours. The main cellular mechanism of the bronchodilator action of theobromine is the blocking of adenosine receptors [27], resulting in relaxation of smooth muscles in the bronchi of the lung thereby increasing the airway diameter by dilating the bronchi. Theobromine also inhibits phosphodiesterases, but this effect only happens at very high amounts of theobromine that people normally did not consume in a typical diet. High doses of theobromine should not be consumed due to unwanted side effects. For example, a dose of 350 mg theobromine may lead to increased heart rate in normal adults and a dose of 1000 mg may result in nausea and vomiting [28].

The PEF is an established criterion for assessing the ventilatory capacity of the lung. It presents a circadian rhythm and briefly increases after administration of theobromine. Further knowledge is needed on the influence of early and late chronotypes and the bronchodilator effect of theobromine on the 24-h pattern of PEF. Previous studies investigated the diurnal variation of PEF in healthy humans, asthmatics and patients with respiratory diseases [10,11,29], but did not consider in their studies the chronotype of study participants. Knowledge of differences between chronotypes in the 24-h variation of PEF may contribute to a better understanding of the relationship between pulmonary function and circadian rhythm of PEF and may help to determine an individual’s time of best performance. Furthermore, most ambulatory studies conducted so far examined the variation in PEF during waking hours but did not include PEF nocturnal readings during sleep. Recording PEF during sleeping hours is of particular interest because sleep and the circadian system independently affect the PEF [30]. A further question to be answered is whether the bronchodilator theobromine influences the morning increase differently compared to the evening decrease of PEF in healthy humans and whether this effect differs between chronotypes. Therefore, this study was designed to investigate two questions. The first question is whether the circadian variation in PEF differs between early and late chronotypes. Previous studies showed that early chronotypes exhibit an earlier timing in sleep-wake cycle and in circadian markers for temperature and melatonin, when compared to late chronotypes [15]. The first hypothesis of this study states that early chronotypes will have their circadian maximum for PEF at an earlier time than late chronotypes. The hypothesis is based on the finding that the temporal distribution of individual acrophases for PEF is bimodal among normal individuals [14] indicating the presence of two groups with different timing for maximum PEF. To test the hypothesis PEF was recorded at 4-hour intervals over a period of 24 hours, including measurements during sleeping hours, in healthy samples of early and late chronotypes, and the acrophases of both chronotypes were compared for differences.

The circadian rhythm for PEF shows a rise in the morning and a decline in the evening [7,8]. The administration of theobromine, due to its bronchodilator effect, will therefore strengthen the morning rise in PEF and weaken the evening decline in PEF. The second question of this study is whether the bronchodilator effect of theobromine on PEF differs between chronotypes. The second hypothesis of this study states that theobromine will have a different effect on the morning rise and evening decline of PEF in early and late chronotypes, if they differ in their acrophases for PEF. If the morning rise begins later in late chronotypes relative to early chronotypes, then the administration of theobromine in the morning will more strongly affect the morning rise in PEF in late chronotypes than in early chronotypes. If the evening decline in PEF starts earlier in early chronotypes relative to late chronotypes, then the administration of theobromine in the evening will have a greater impact on the evening decline in PEF in early chronotypes than in late chronotypes. To test this hypothesis, the morning rise in PEF as well as the evening decline in PEF was compared in healthy early and late chronotypes on days with theobromine administration in the morning or in the evening vs. days without theobromine administration.

## Materials and Methods

### Participants

Participants were 97 healthy female volunteers (54 early chronotypes and 43 late chronotypes), aged 18–35 years, who satisfied the criteria for chronotype, set by the morningness-eveningness questionnaire by Horne and Östberg [31], German validated version by Griefahn et al. [32]. High scores indicate early chronotypes and low scores late chronotypes. Mean age ± standard deviation was for early chronotypes 24.39 ± 3.68 years and for late chronotypes 24.33 ± 3.72 years. The study was restricted to young adult females, because age and sex are known to influence PEF values [1]. Circadian rhythm-related inclusion criteria were presence of a regular sleep-wake pattern, a sleep time between six and nine hours, no use of exogenous melatonin, absence of shift work or jet lag within the past three months prior to the study, as assessed by questionnaire. Health-related exclusion criteria were a history of asthma, chronic bronchitis, COPD, allergy, infection of upper and lower respiratory tract, presence of other chronic disorders, acute illness and use of medication. Health status was assessed by medical history. The participants were non-smokers, did not present obesity, were not dieting, pregnant or lactating and did not competitive sports. They signed informed consent forms to participate in the study. The ethics committee at the Medical Faculty of the Christian-Albrechts-University, Kiel, approved the study protocol (D 575/22). The study has been performed in accordance with the Declaration of Helsinki.

### Procedure

All participants were personally informed about the study procedure at the Department of Human Biology at the university in Kiel and received additional written instructions to take home. They were instructed to follow a regular sleep-wake pattern keeping bedtimes according to their biological rhythms starting three days prior to data collection and to continue during the study days. They got data sheets to note their bedtimes, were equipped with peak flow meters, were carefully trained in recording PEF and took test measurements before starting the study. On the study days, they recorded at home their PEF over a 24-h sampling period at 4-h intervals at 08:00, 12:00, 16:00, 20:00, 24:00, 04:00 and 08:00 h. These time points were chosen because a laboratory study demonstrated in healthy individuals a minimum PEF around 04:00 h and a maximum PEF around 16:00 h [7]. For recording PEF at night, participants set an alarm clock to wake up, stood up, measured their PEF and continued to sleep. Waking for a few minutes did not affect the pattern of sleep cycles [33]. During collection of data, the participants followed their normal daily routines but were instructed to avoid consuming chocolate and caffeinated drinks and daytime napping. Within one hour before recording PEF they should refrain from doing exercise.

In a subgroup of 44 participants (26 ET and 18 LT), the effect of 48 mg theobromine on PEF was examined. The theobromine subgroup consisted of women who agreed to participate in the theobromine study. Theobromine was orally administered as 40 g dark chocolate with 71% cocoa content (Vivani “Feine Bitter”, EcoFina GmbH, Herford, Germany), because the absorption of theobromine from chocolate is more rapid than from capsules leading to higher maximum theobromine blood plasma concentrations [25]. A quantity of 40 g dark chocolate was used because it represents a usual amount consumed and no side effects are to be expected. Very high doses of theobromine may increase heart rate, produce restlessness, trembling, sweeting, headache, nausea and vomiting [34]. The study period extended over seven days. Days 1–3 served to stabilize the PEF rhythms and the participants took test measurements with the peak flow meter. On days 4, 5 and 7, the participants recorded their PEF over a 24-h period at 4-h intervals at 08:00, 12:00, 16:00, 20:00, 24:00, 04:00 and 08:00 h. On day 4, there was no administration of theobromine because this day served as control (baseline). A quantity of 48 mg theobromine was administered on day 5 in the morning at 10:00 h and on day 7 in the evening at 22:00 h. Theobromine was not administered on day 6 so that there was more than a 24-h time interval between the two days with theobromine administration. During data collection, the participants refrained from consuming chocolate, cocoa and caffeinated drinks and daytime napping. They did no exercise within one hour before recording PEF values. The participants noted their bedtimes in sleep diaries (days 1–3) or data sheets (days 4–7) and times were verified by actigraphy. They further kept a dietary record to ensure that they did not consume foods and drinks containing theobromine and caffeine. For this purpose, the participants noted in their dietary records the quantities of all foods and drinks that they consumed during the study days as well as the times of consumption.

### Assessment of anthropometric, sleep-wake and nutritional characteristics

Body weight and body height were measured using an electronic scale (TGF 302H, Rossmann, Burgwedel) and a wall-mounted measuring device (Seca 206, Seca, Hamburg), respectively. The body mass index (BMI) was calculated as weight in kilograms divided by height in meters squared. All participants got data sheets to note their bedtimes. The participants of the theobromine subgroup additionally completed a sleep-wake diary (days 1–3), wore an accelerometer to objectively verify bedtimes (days 4–7) and completed a dietary record to ensure that no foods and drinks were consumed that contain theobromine or caffeine (days 4, 5, 7). The participants used the sleep-wake diary of the German Sleep Society (DGSM [35]), where they noted bedtimes, lights-off time before sleep in the evening and wake-up time in the morning as well as periods of wakefulness during night. They wore on their non-dominant upper arm the SenseWear® Armband MF-SW (SWA; Bodymedia, Pittsburgh, Pennsylvania, USA) that uses a triaxial accelerometer and were instructed to press an eventmarker on the device when they turned off the light to sleep in the evening and again when they awoke in the morning. Thereafter, they returned the SWA, data was transferred on a computer and scored using the SenseWear Professional 8.0 software of the manufacturer. The SWA allows valid and reliable measures of physical activity [36]. Data from the dietary record was analyzed using the software PRODI® 7 expert, version 7.1 (Nutri-Science GmbH, Freiburg, Germany).

### Determination of PEF

The PEF was recorded using the portable asmaPLAN+ peak flow meter (Vitalograph, Maids Moreton Buckingham, England). The measuring range of this device is 50–800 L/min, scale intervals are 10 L/min below 700 ml and 20 L/min above 700 ml. According to the manufacturer, accuracy is <10%, repeatability <5% or 10 L/min and inter-instrument variation <5% or 10 L/min. In the subgroup with theobromine administration, the PEF was determined using the digital asma-1™ peak flow meter, model 4000 (Vitalograph, Maids Moreton Buckingham, England) that has an electronic respiratory monitor, a measuring range of 25–840 L/min and an accuracy <10%. Each participant received a new peak flow meter, was individually instructed and trained for recording her own PEF. A trained person explained and demonstrated the maneuver to measure the PEF and thereafter the participant performed test measurements under supervision of the trained person. All PEF readings were taken in indoor rooms with the participant standing upright and holding the peak flow meter in a horizontal position. The participant reset the peak flow meter to the zero mark, took a deep breath, put the mouthpiece of the instrument in the mouth, sealed the lips around the mouthpiece and blow out as fast and forcefully as possible in one blow. The participant was instructed to avoid blocking the mouthpiece with the tongue, should make sure that there was no air leak between lips and mouthpiece of the peak flow meter and should avoid preventing the pointer of the device by fingers from moving when breathing into the peak flow meter. The participant received a data sheet for taking home to record the PEF values. At each time point, three PEF readings were obtained, which should be close together and the highest reading was used for the analyses. For PEF recordings during the night, each participant was carefully instructed to set an alarm clock to wake up, and then perform the three measurements in standing position. The participants of the theobromine subgroup additionally wore a triaxial accelerometer (SWA-MF) to control for their physical activity at night and to control for potential disturbances in sleep that might affect the PEF recordings. However, waking for a few minutes to perform the PEF recordings did not affect the pattern of sleep cycles [33].

### Statistical analyses

Data analysis was performed using SPSS software for Windows version 29.0 (IBM, Armonk, NY, USA). Values are presented as means ± standard deviation (SD) or standard error of the mean (SEM). Normal distribution of data was tested using the Shapiro-Wilk test. Differences between early and late chronotypes in general characteristics and PEF readings were analyzed using Student’s t-tests or Mann-Whitney-u-tests, where appropriate. Differences in PEF readings between days with vs. without administration of theobromine were tested for significance using paired sample’s t-tests or Wilcoxon tests, depending on normal distribution of data. A one-way repeated measures analysis of variance (ANOVA) was conducted for each chronotype to test whether there are statistically significant differences in PEF values over the seven time points (08:00, 12:00, 16:00, 20:00, 24:00, 04:00 and 08:00 h) of the 24-h day. This allows testing for significant 24-h variation in PEF recordings. The dependent variable was PEF and the independent variable was “time”. The null hypothesis states that the PEF value is the same at all seven time points, because the means are equal. The alternative hypothesis is that the PEF value is significantly different at one or more time points. Sphericity is an assumption of the repeated measures ANOVA. Sphericity means a condition of equal variances among the differences between all possible pairs of levels of the independent variable. Mauchly’s test of sphericity was applied to evaluate whether the sphericity assumption has been violated. Since this was the case, the Greenhouse-Geisser correction was performed to adjust for lack of sphericity. In order to determine the strength of the effect of the independent variable (time) on the dependent variable (PEF), effect sizes were calculated using Cohen’s index f = \[
\sqrt {{\textstyle{{\eta ^2} \over {1\;{-}\;\eta ^2}}}}
\] , where \[
{\eta ^2}\;
\] means partial eta-squared. Partial eta-squared expresses the amount of variance accounted for by the independent variable “time”. For ANOVA, Cohen [37] suggests that f = 0.10 indicates a small effect size, f = 0.25 a medium effect size and f = 0.40 a large effect size. Parameters of 24-h variation in PEF, which were compared between early and late chronotypes, were mean 24-h PEF, minimum PEF, maximum PEF, clock time of maximum PEF and amplitude % mean of PEF. They were determined over the whole 24-h period for each participant and then averaged by chronotype. The amplitude % mean of PEF was calculated for each individual as the difference between the highest and lowest 24-h reading, divided by mean 24-h reading and multiplied with 100 [38]. The relationship of time of maximum PEF with chronotype score and bedtimes was analyzed using Pearson or Spearman rank correlation coefficients. Statistical tests were performed two-sided and a *P*-value of less than 0.050 was considered statistically significant.

## Results

### Comparison of 24-h variation of PEF in early and late chronotypes

The mean chronotype score for early chronotypes and late chronotypes was 65.4 (range 59.0–80.0) and 37.7 (range 25.0 to 41.6), respectively. There were 11 definitely and 43 moderately early chronotypes and 39 moderately and 4 definitely late chronotypes. Table 1 summarizes for each chronotype anthropometric characteristics and bedtimes on the study day. Early and late chronotypes did not significantly differ in age and anthropometric parameters. Awakening times and sleep onset times were significantly advanced by 77 min and 86 min, respectively, in early chronotypes compared to late chronotypes.

**Table 1:** Anthropometric characteristics, bedtimes and peak expiratory flow rate (PEF) in early and late chronotypes.


PARAMETER	EARLY CHRONOTYPES (n = 54) MEAN ± SD	LATE CHRONOTYPES (n = 43) MEAN ± SD	GROUP COMPARISON *p* VALUE

Age (year)	24.39 ± 3.68	24.33 ± 3.72	0.804

Body weight (kg)	66.31 ± 9.68	67.34 ± 11.37	0.791

Body height (m)	1.69 ± 0.05	1.70 ± 0.07	0.238

BMI (kg/m²)	23.17 ± 2.69	23.13 ± 3.29	0.836

Awakening time (hh:mm)^a^	06:52 ± 66.36	08:09 ± 70.73	<0.0001

Sleep onset time (hh:mm)^a^	22:39 ± 50.41	24:05 ± 63.57	<0.0001

PEF 24-h mean (L/Min)	422 ± 75	419 ± 65	0.708

PEF 24-h minimum (L/Min)	386 ± 80	379 ± 70	0.668

PEF 24-h maximum (L/Min)	452 ± 70	456 ± 70	0.611

PEF amplitude % mean	16.54 ± 8.82	19.10 ± 12.83	0.586

PEF time of max. (hh:mm)^a^	13:21 ± 261	16:26 ± 291	0.003


BMI = body mass index, max. = maximum, SD = standard deviation. ^a^ SD is given in minutes.

**Figure 1:**
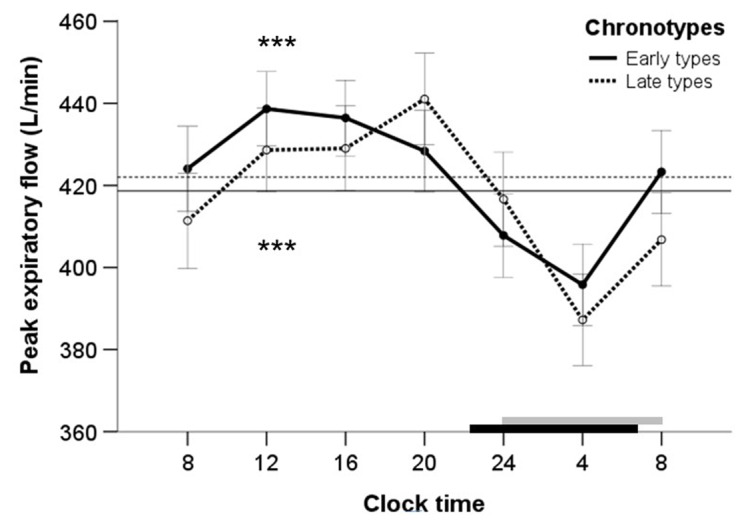
The 24-h variation of mean peak expiratory flow rate (PEF), measured at 4-h intervals (8, 12, 16, 20, 24, 4 and 8 h), in 54 early chronotypes (solid line) and 43 late chronotypes (dotted line). Each data point represent mean ± SEM. Horizontal dark line (early chronotypes) and dotted line (late chronotypes) indicate the 24-h mean PEF. Horizontal black bar (early chronotypes) and gray bar (late chronotypes) indicate mean sleeping times. Asterisks show significance level for the 24-h variation in PEF (repeated measures ANOVA), *** *P* < 0.001.

The analysis of variance with repeated PEF measurements showed that the independent variable “time” had a significant effect on the 24-h variation in PEF. This was true for early chronotypes [Greenhouse-Geisser F(4.41, 233.80) = 29.17, *P* < 0.0001, Figure 1] and late chronotypes [F(4.44, 186.56) = 18.60, *P* < 0.0001, Figure 1]. It shows that both chronotypes exhibited a significant 24-h variation in PEF. Partial eta² expresses the amount of variance in PEF accounted for by the independent variable “time”. Partial eta² was 0.36 in early chronotypes and 0.31 in late chronotypes. This means that the independent variable time explained 36% of variance of PEF in early chronotypes and 31% of variance of PEF in late chronotypes. From partial eta² the effect size f was calculated. The effect size describes the strength of the effect of the independent variable “time” on the dependent variable PEF. The effect size was 0.75 for early chronotypes and 0.67 late chronotypes. This means that the variable “time” had a large effect on PEF in each chronotype, because f was >0.40.

**Figure 2:**
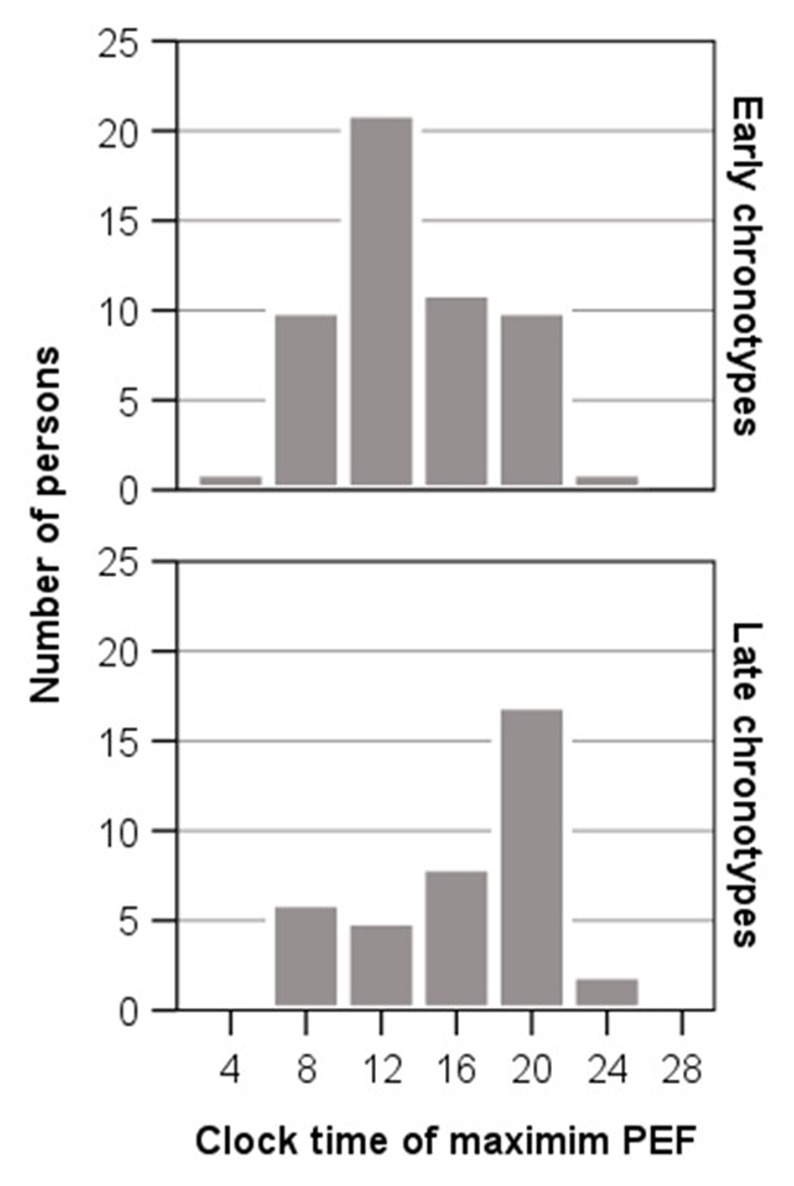
Histogram showing temporal distribution of individual clock times for maximum PEF of 54 early and 43 late chronotypes over the 24-h period. Early and late chronotypes show a different distribution of clock times for maximum PEF. Maximum PEF was most common between 12:00 h and 16:00 h in early chronotypes and between 16:00 h and 20:00 h in late chronotypes.

Figure 1 presents the temporal pattern of the 24-h variation in PEF for each chronotype showing mean values for PEF at the seven time points of the 24-h period. Early chronotypes exhibited a gradual increase in mean PEF from early morning before reaching a maximum at 12:00 h and thereafter displayed a progressive fall throughout the afternoon and evening to a minimum at 04:00 h at night. The mean PEF of late chronotypes raised from the morning throughout the afternoon before reaching the maximum at 20:00 h and then exhibited a gradual decrease throughout the evening to the minimum at 04:00 h. The highest mean PEF occurred in both chronotypes during waking hours and the lowest mean PEF during sleeping hours. The difference between PEF values, observed at the time points with the highest and the lowest mean PEF, was significant for each chronotype (early chronotypes, mean difference ± SD between 12:00 h and 04:00 h: 42.86 ± 31.56 L/min, t = 9.98, *P* < 0.0001; late chronotypes, difference between 20:00 h and 04:00 h: 53.83 ± 47.35 L/min, *P* < 0.0001). Figure 2 displays the distribution of the individual clock times for maximum PEF, separately by chronotype. The individual distribution of clock times for maximum PEF differs between early and late chronotypes. The maximum PEF was most common between 12:00 h and 16:00 h in early chronotypes and between 16:00 h and 20:00 h in late chronotypes.

For comparing parameters of PEF variation between early and late chronotypes, the 24-h mean PEF, minimum PEF, maximum PEF, amplitude % mean of PEF and time of maximum PEF were determined for each individual over the 24-h period and then mean values were calculated (Table 1). Significant differences between chronotypes were only present for mean time of maximum PEF that was at 13:21 h in early chronotypes and at 16:26 h in late chronotypes. Thus, the 24-h maximum for PEF occurred approximately three hours earlier in early chronotypes than in late chronotypes (*P* = 0.003). The time of the 24-h PEF maximum correlated negatively and significantly with the chronotype score (rs = –0.34, *P* = 0.001), meaning that the 24-h maximum for PEF occurred at earlier clock time with increasing morning preference (higher chronotype score). In addition, the time of the 24-h PEF maximum correlated positively and significantly with the bedtimes of the participants, meaning that earlier bedtimes were related to earlier times of the 24-h maximum in PEF. There was a stronger relationship with the awakening time (rs = 0.40, *P* = 0.00015) than with the sleep onset time (rs = 0.27, *P* = 0.012).

### Effect of theobromine on the 24-h variation of PEF in both chronotypes

The effect of theobromine on the 24-h variation in PEF was analyzed in a subgroup of 44 chronotypes. Table 2 summarizes anthropometric characteristics and bedtimes for the three study days in the early and late chronotypes. They did not significantly differ in anthropometric characteristics. Early chronotypes, compared to late chronotypes, had significantly earlier awakening times and sleep onset times on each of the three study days.

**Table 2:** Anthropometric characteristics, bedtimes and peak expiratory flow rate (PEF) on day 4 (baseline, no theobromine), day 5 (48 mg theobromine at 10:00 h) and day 7 (48 mg theobromine at 22:00 h) in early and late chronotypes.


PARAMETER	EARLY CHRONOTYPES (n = 26) MEAN ± SD	LATE CHRONOTYPES (n = 18) MEAN ± SD	GROUP COMPARISON *p* VALUE

Age (year)	25.65 ± 3.78	24.72 ± 4.18	0.446

Body weight (kg)	63.42 ± 6.60	64.61 ± 10.20	0.667

Body height (m)	1.68 ± 0.04	1.70 ± 0.05	0.159

BMI (kg/m²)	22.49 ± 2.20	22.31 ± 3.07	0.817

Awakening time (hh:mm)^a^			

Day 4	06:48 ± 66.58	07:54 ± 71.79	0.003

Day 5	07:14 ± 66.36	08:09 ± 70.73	0.029

Day 7	07:13 ± 65.22	08:05 ± 61.54	0.007

Sleep onset time (hh:mm)^a^			

Day 4	22:53 ± 51.66	23:55 ± 49.20	<0.001

Day 5	23:03 ± 58.27	24:10 ± 51.82	<0.001

Day 7	23:14 ± 55.96	24:11 ± 51.49	0.001

PEF 24-h mean (L/Min)			

Day 4 #	460 ± 60	429 ± 77	0.138

Day 5 #	458 ± 66	434 ± 81	0.280

Day 7 #	458 ± 66	430 ± 75	0.201

PEF amplitude % mean			

Day 4 #	14.28 ± 5.42	18.13 ± 14.65	0.667

Day 5 #	14.62 ± 7.42	15.90 ± 11.23	0.981

Day 7 #	14.92 ± 8.05	13.65 ± 8.04	0.568


BMI = body mass index, SD = standard deviation. ^a^ SD is given in minutes. # There were no significant differences between day 4 vs. day 5 or between day 4 vs. day 7 in 24-h mean PEF nor in amplitude % mean PEF in any chronotype.

**Figure 3:**
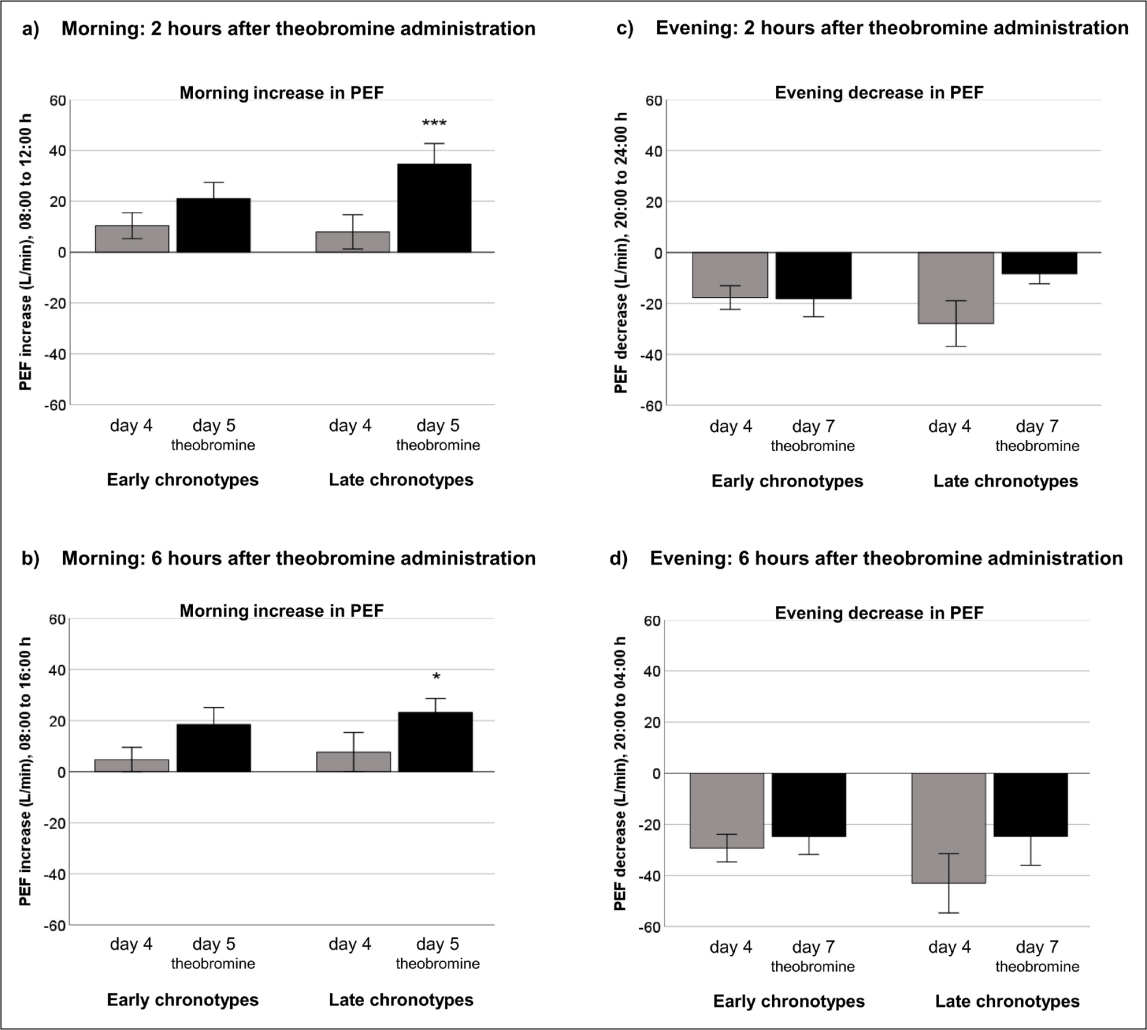
Effect of 48 mg theobromine on morning increase and evening decrease in peak expiratory flow rate (PEF) **(a)** two hours and **(b)** six hours after administration in the morning as well as **(c)** two hours and **(d)** six hours after administration in the evening, separately for early and late chronotypes. Compared to baseline (day 4, no theobromine, gray bars), the administration of theobromine at 10:00 h in the morning (day 5, black bars) strengthened the morning increase of PEF in both chronotypes, which was more pronounced and only significant in late chronotypes. The administration of theobromine at 22:00 h (day 7, black bars) weakened the evening decrease of PEF in late chronotypes, but did not affect it in early chronotypes. * *P* < 0.050, *** *P* < 0.001.

We first examined in both chronotypes the short-term effect of 48 mg theobromine on PEF over time periods of two and six hours following administration of theobromine. We were interested how morning administration of theobromine would change the morning increase of PEF and how evening administration of theobromine would influence the evening decrease in PEF. Figure 3 shows the effect of 48 mg theobromine on the morning increase in PEF, two hours (Figure 3a) and six hours (Figure 3b) after administration, separately for each chronotype. The administration of theobromine on day 5 at 10:00 h in the morning, compared to baseline (day 4, no theobromine), resulted in both chronotypes in a stronger morning increase in PEF over the next two hours than at baseline (Figure 3a). The increase was more pronounced in late chronotypes (day 4 vs. day 5, mean ± SEM: 7.94 ± 6.72 L/min vs. 34.61 ± 8.07 L/min, *P* = 0.004) than in early chronotypes (10.42 ± 5.10 L/min vs. 21.08 ± 6.39 L/min, *P* = 0.166) and was only significant in late chronotypes. Even six hours after administration (Figure 3b), the effect of theobromine on PEF was still present in late chronotypes and significant, compared to baseline (day 4 vs. day 5, 7.61 ± 7.69 L/min vs. 23.11 ± 5.49 L/min, *P* = 0.049), albeit less pronounced than two hours after administration.

Figure 3 also displays for each chronotype the effect of 48 mg theobromine on the evening decrease in PEF, two hours (Figure 3c) and six hours (Figure 3d) after administration. Administration of theobromine in the evening at 22:00 h on day 7 resulted in late chronotypes in a weaker evening decrease in PEF over the next two hours, compared to baseline, which was near the limit of significance (day 4 vs. day 7, –27.53 ± 9.50 L/min vs. –8.47 ± 3.79 L/min, *P* = 0.063, Figure 3c). By contrast, evening administration of theobromine had no effect on PEF in the early chronotypes (–17.69 ± 4.66 L/min vs. –18.15 ± 7.03 L/min, *P* = 0.961). Six hours after administration the effect of theobromine on PEF remained non-significant in both chronotypes (early chronotypes: –29.33 ± 5.43 L/min vs. 24.88 ± 6.84 L/min, *P* = 0.633; late chronotypes: –43.06 ± 11.60 L/min vs. –24.73 ± 11.34 L/min, *P* = 0.055, Figure 3d).

We further analyzed whether administration of theobromine influences the 24-h variation in PEF. For this, we compared the 24-h mean PEF and amplitude % mean of PEF over the 24-h period on days with and without administration of theobromine. Table 2 displays the 24-h mean PEF and the PEF amplitude % mean for day 4 (no theobromine, baseline), day 5 (theobromine at 10:00 h) and day 7 (theobromine at 22:00 h) by chronotype. Compared to baseline, neither morning nor evening administration of 48 mg theobromine affected the 24-h mean PEF in any chronotype (early chronotypes, day 4 vs. day 5, mean ± SD: 460 ± 60 L/min vs. 458 ± 66 L/min, *P* = 0.545; day 4 vs. day 7: 460 ± 60 L/min vs. 458 ± 66 L/min, *P* = 0.540; late chronotypes, day 4 vs. day 5: 429 ± 77 L/min vs. 434 ± 81 L/min, *P* = 0.217; day 4 vs. day 7; 429 ± 77 L/min vs. 430 ± 75 L/min, *P* = 0.784; t tests). The amplitude % mean PEF was on both days with theobromine administration, compared to baseline, non-significantly higher in early chronotypes (day 4 vs. day 5, *P* = 0.301; day 4 vs. day 7, *P* = 0.454, Table 2) and non-significantly lower in late chronotypes (day 4 vs. day 5, *P* = 0.711; day 4 vs. day 7, *P* = 0.076).

## Discussion

### Effect of morningness-eveningness preference on the 24-h variation of PEF

The present study provided for the first time 24-h profiles for PEF from early and late chronotypes. Both chronotypes displayed significant 24-h variation for PEF. We found that the mean time of maximum PEF was significantly advanced by approximately three hours in early chronotypes (13:21 h) compared to late chronotypes (16:26 h). Thus, we could confirm the hypothesis that early chronotypes have their circadian maximum for PEF at an earlier time than late chronotypes. The time difference of three hours for maximum PEF between chronotypes agrees with studies showing that the circadian phase markers for temperature and melatonin occur 2–3 hours earlier in early chronotypes than in late chronotypes [15]. This is in line with the negative and significant correlation observed between chronotype score and time of maximum PEF, indicating that a higher morningness preference was related to an earlier timing of maximum PEF and *vice versa*. This also fits with the positive and significant relationship observed between maximum PEF and bedtimes meaning that earlier awakening and sleep-onset times are related to an earlier time of maximum PEF. The awakening and sleep onset times were significantly advanced in early compared to late chronotypes that is consistent with previous findings [39].

The individual times for maximum PEF showed different temporal distributions in early and late chronotypes. A pooling of the individual times for maximum PEF from both chronotypes into one group would therefore result in a bimodal distribution. This could explain findings of a previous study that individual acrophases for PEF displayed a bimodal distribution in normal subjects [14]. The bimodal distribution might be attributed to the different timing of maximum PEF in different chronotypes.

The mean times for maximum PEF observed in this study in early and late chronotypes are within the wide range of 12:48 h to 16:26 h for mean acrophases for PEF reported in the literature [7,8,10,40]. Because previous studies did not differentiate between chronotypes, different proportions of early and late chronotypes within the study groups may partly explain differences between studies in the timing of acrophase. The results of the present study therefore support the role of morningness-eveningness-preference in determining the time of maximum PEF.

The findings of this study in healthy individuals may also apply to patients with asthma, because mean acrophases of circadian PEF rhythms observed in normal individuals did not significantly differ from those in asthmatic individuals [7]. This implies that knowing about the individual chronotype might help to determine the optimal time for therapy in asthmatics. Scheer et al. [17] already suggested that the internal biological time should be considered for optimal therapy in patients with asthma. The determination of chronotype may be relevant because late chronotypes were more frequently associated with respiratory symptoms than early chronotypes in a group of adolescents [41]. Differences between chronotypes were also found in adult asthmatics showing that early chronotypes had a lower prevalence of nocturnal symptoms than intermediate chronotypes [42]. The present findings may therefore have clinical relevance regarding chronotherapy for respiratory diseases that affect airways (asthma, COPD, fibrosis). Chronotherapy considers the circadian rhythm of patients for a better therapy outcome. Coordinating the timing of administration of medication with circadian rhythms would allow modulating pharmacokinetics of drugs to optimize their action, reduce their dosage and minimize unwanted side effects. The differences observed in this study in the 24-h timing of PEF between chronotypes argue for a potential benefit in a personalized chronotherapeutic approach to respiratory diseases. The finding of an earlier circadian phase for PEF in early chronotypes, compared to late chronotypes, suggests that optimal drug timing might be earlier for early chronotypes than for late chronotypes to enhance treatment efficacy. In addition, we demonstrated that late chronotypes responded more strongly to the administration of the bronchodilator theobromine in the morning than early chronotypes. This suggests that morning dosing of bronchodilator drugs might confer better protection against respiratory diseases in late chronotypes than in early chronotypes or that the dosage could be reduced in late chronotypes. Future studies are needed to investigate whether the chronotype of patients modifies the effect of drugs against respiratory diseases and whether the timing of drugs according to the chronotype of the patient can enhance its efficacy. The identification of chronotype can be based on the morningness-eveningness score (MEQ score) of the Horne and Östberg questionnaire [31]. In addition, an individual 24-h profile for PEF recordings may help to determine the time of maximum and minimum PEF for optimizing timing of therapy. Finally, the disruption of circadian rhythms (chronodisruption) should also be taken into account. Chronodisruption leads to the progression of respiratory diseases and it is more evident in late chronotypes who more often show a discrepancy between biological and social time (social jetlag) than early chronotypes.

This study observed no differences between chronotypes in absolute values for maximum PEF, minimum PEF, 24-h mean and 24-h variation in PEF (amplitude % mean). This indicates that parameters of 24-h variation of PEF did not differ between chronotypes. The amplitude of variation observed in this study was higher than that previously described in normal adults. This can be explained by the design of the present study that considered PEF readings over the whole 24-h period including PEF readings from sleeping hours at nighttime, where participants awoke briefly from sleep to record their PEF. In contrast, previous ambulatory diurnal studies restricted PEF recordings to waking hours [8,9,10,12]. The inclusion of PEF readings from sleeping time is relevant, because the overnight decline in PEF is stronger when sleep occurred compared to an overnight decline when sleep does not occur [30]. In addition, a laboratory study demonstrated the importance of sleep in the genesis of diurnal variation in PEF [43]. Consistent with that, Jindal et al. [44] showed that the PEF amplitude % mean was considerably less than the true variability, if PEF was measured at conventional times, excluding measurements at 04:00 h and 16:00 h. Moreover, the present study was conducted in females, who generally have higher amplitudes % mean PEF than males [45,46]. Therefore, PEF readings from sleeping hours should be included when the 24-h variation of PEF is examined under natural living conditions.

### Effect of theobromine on the 24-h variation in PEF in both chronotypes

A further question to be answered was how the bronchodilator theobromine affects the morning increase as well as the evening decrease in PEF in early and late chronotypes. A bronchodilator dilates the bronchi and bronchioles thereby decreasing the resistance in the airways resulting in increased airflow through the lung. We therefore expected that morning administration of theobromine would strengthen the morning increase in PEF and that evening administration would weaken the evening decrease in PEF. We stated the hypothesis that the administration of theobromine in the morning will more strongly affect the morning rise in PEF in late chronotypes than in early chronotypes, if late chronotypes have a later acrophase for PEF. Our findings confirmed the hypothesis. Results showed that administration of 48 mg theobromine in the morning lead to a stronger morning increase in PEF in both chronotypes, but the effect of theobromine on PEF was more pronounced and only significant in late chronotypes. Administration of theobromine in the evening weakened non-significantly the evening decline in PEF in late chronotypes, but had no effect in early chronotypes. These findings provide therefore evidence, firstly, that theobromine may have a stronger bronchodilator effect on PEF in the morning than in the evening and secondly, that theobromine differently affects PEF in early and late chronotypes.

The stronger morning than evening effect of theobromine on PEF may be attributed to endogenous circadian processes and varying external behavioral influences on bronchodilation throughout the day. Circadian changes in airway physiology, autonomous nervous system and pharmacokinetics of theobromine may lead to time-dependent alterations in airway responsiveness. Goyal et al. [40] demonstrated circadian changes in the autonomous nervous system and its relationship with circadian changes in PEF.

Circadian variation in pharmacokinetics has been reported in healthy volunteers for the vasodilator pentoxifylline [47] that is just like theobromine a methylxanthine derivate. Here, the maximum plasma concentration was significantly lower after drug administration at 10:00 h in the morning than at later times of the day. Earlier studies demonstrated circadian rhythms in pharmacokinetics of drugs and elucidated some underlying mechanisms [48]. Circadian changes in the pharmacokinetics of theobromine might therefore explain some of the observed effects of chronotype and theobromine on PEF. Pharmacokinetic processes influenced by circadian regulation are absorption, distribution, metabolism and excretion of theobromine. The absorption of orally administered theobromine into the systemic circulation depends in part on gastrointestinal motility and blood flow. Known rhythmic changes in gastrointestinal motility and blood flow [49] might therefore contribute to a temporal variation in theobromine absorption. For theophylline that is, like theobromine, a methylxanthine, diurnal variation in absorption following oral administration was reported [50]. Theobromine is metabolized in the liver into xanthine by a hepatic cytochrome P450 enzyme and subsequently into methyluric acid by xanthine oxidase [51]. The expression of cytochrome P450 genes is regulated by the biological clock [52]. The circadian variation in theobromine metabolizing enzymes, including the hepatic p450 system, may thus alter the availability of theobromine at different times in a day. In addition, the hepatic blood flow follows a circadian rhythm [53]. This may result in a time-dependent hepatic clearance of theobromine. The major metabolites of theobromine in human urine are methylxanthine (54–68%) and methyluric acid (7–12%) [54]. The urinary excretion of theobromine metabolites will be time-dependent, because all processes in the kidney (glomerular filtration, tubular reabsorption and tubular secretion) show circadian rhythms [55]. In summary, circadian changes in absorption, metabolism and excretion of theobromine might explain the differences between morning and evening effects of theobromine on PEF.

In addition, it is well known that theobromine acts through blocking of adenosine receptors [27] that causes relaxation of smooth muscles in the airways of the respiratory tract. A further explanation could therefore be a time-dependent sensitivity of adenosine receptors to theobromine. Evidence for this assumption comes from an animal study that found a circadian variation for adenosine receptors in the brain of mice [56]. Furthermore, epinephrine acts like theobromine as a bronchodilator. Plasma levels of epinephrine fluctuate in a circadian manner showing nadir (04:00 h) and peak (16:00 h) at similar times as theobromine in healthy and asthmatic subjects [57]. However, confounding external factors such as changes in behavior and environment throughout the day makes it difficult to isolate the pure circadian influence of theobromine on PEF from external factors. Exercise, daytime napping and consumption of foods and drinks that contain theobromine and caffeine can briefly induce bronchodilation and thus may affect PEF readings [22,23,24]. Although we controlled for these factors in the present study by instructing the participants to avoid exercise, napping and special foods/drinks on study days and by verifying this by actigraphy and dietary records, such effects could not be excluded in an ambulatory study. Thus, there is a need for laboratory studies applying a constant routine procedure that would help to elucidate in early and late chronotypes the circadian influence of theobromine on PEF independent of external influences.

We further analyzed whether the administration of 48 mg theobromine differently effects PEF in early and late chronotypes. It was expected that morning administration of theobromine would have a smaller effect on PEF in early chronotypes than in late chronotypes, because early chronotypes reach their maximum PEF at earlier times than late chronotypes. As expected, we found a stronger morning increase in PEF in late chronotypes. The administration of theobromine at 10:00 h in the morning induced a significant bronchodilation in late chronotypes, as evidenced by a fourfold increase (436%) in PEF until 12:00 h, when this PEF value was compared to the value obtained at baseline, while the increase in early chronotypes was only half as high (202%). The smaller and non-significant increase of PEF after administration of theobromine in early chronotypes could be explained by the phenomenon that their PEF values were already at higher levels at the time of theobromine administration than the PEF values of late chronotypes. The PEF of late chronotypes was significantly increased at two and six hours after administration and was highest at two hours after administration. This confirms previous results showing that the peak bronchodilation occurred two hours after administration of theobromine and that the bronchodilation lasted for six hours [23]. It is also in line with earlier results reporting that peak concentrations of theobromine absorption from chocolate occurred in blood plasma approximately two hours after administration [25].

We further stated the hypothesis that the administration of theobromine in the evening will weaken more strongly the evening decline in PEF in early chronotypes than in late chronotypes, due to the earlier diurnal decline of PEF in early chronotypes. The present results did not confirm this hypothesis. Contrary to the expectation, theobromine administration at 22:00 h in the evening had no effect on PEF in early chronotypes, but weakened non-significantly the evening decline in PEF in late chronotypes (*P* = 0.063). Two and six hours after theobromine administration no significant effect on PEF was found in late chronotypes, although the magnitude of evening decrease of PEF was smaller compared to baseline. It can be assumed that differences between early and late chronotypes in the timing of circadian processes may result in a different responsiveness to theobromine at specific clock times. In addition, differences between chronotypes in timing of daily routines and physical activity, that may interfere with circadian changes in airway resistance, will contribute to the present results. The observed differences between chronotypes in PEF should be further investigated by controlling for external factors. Laboratory studies in early and late chronotypes would help to assess the effect of theobromine on circadian changes in PEF independent of external influences.

Neither morning nor evening administration of 48 mg theobromine, compared to baseline, did change the 24-h mean and amplitude % mean of PEF in any chronotype. This implies that consuming usual amounts (40 g) of dark chocolate with 71% of cocoa would not affect the 24-h variation in PEF. This finding may generally be relevant for interpreting the effect of bronchodilator substances that occur in foods, drinks and medication on airway resistance in humans. Whether a higher quantity of theobromine would have a stronger effect on PEF should be investigated in future studies. However, one should consider that high doses of theobromine may have unwanted side effects [28].

### Strength and Limitations

This study has strengths and limitations. A first strength refers to the inclusion of PEF measurements from sleeping hours that allows assessing the effect of sleep on PEF. Regarding the effect of theobromine on PEF, a second strength is that the same participants recorded PEF values on days with and without theobromine administration. A further strength is that the participants completed dietary records to ensure that they did not consume foods and drinks containing theobromine and caffeine. This study has also some limitations. One limitation confers to the restriction to female participants. Male participants were not included due to the low number of males who were healthy, early or late chronotypes and who agreed to participate in this study. The restriction to female participants may limit the generalizability of the findings to the entire population, because females have lower absolute PEF values than males [2]. This may be due to sex differences in anatomy and physiology, since females have smaller large conducting airways, which are the main sites of airway resistance [58]. Future research should therefore expand the investigation to include male participants. A second limitation is that no clinical examination of lung function was performed to rule out pulmonary diseases. A third limitation is that the participants recorded their PEF values at home that could not be controlled. However, the objective was to measure the 24-h variation of PEF under natural living conditions and all participants were carefully trained to ensure correct PEF measurements.

## Conclusions

This study provided for the first time information on the relationship between the 24-h variation in airway resistance and morningness-eveningness preference in healthy individuals. The main finding was that early and late chronotypes differ in the timing of their maximum PEF by approximately three hours, but did not show differences in mean values for maximum PEF, minimum PEF, 24-h mean PEF and 24-h variation in PEF. This implies that rather the identification of chronotype than the time of day may be important when determining the maximum performance in pulmonary function. Furthermore, this study assessed the bronchodilator effect of 48 mg theobromine on PEF in healthy early and late chronotypes under ambulatory conditions. The consume of 40 g dark chocolate containing 48 mg theobromine had a stronger bronchodilator effect on PEF in the morning than in the evening and this effect was stronger in late than in early chronotypes. Administration of theobromine did not alter parameters of 24-h variation in neither chronotype. These findings may be generally relevant for interpreting the effect of bronchodilator substances that occur in foods, drinks and medication on airway resistance in humans. Based on these findings future studies should further examine the responsiveness of different chronotypes on theobromine and other bronchodilator substances.

## Data availability statement

The participants of this study did not give written consent for their data to be shared publicly, so due to the sensitive nature of the research supporting data is not available.
